# Primary-school recorded special educational needs in children born with major congenital anomalies in England: A population-based study using the Education and Child Health Insights from Linked Data database

**DOI:** 10.23889/ijpds.v8i2.2340

**Published:** 2023-09-14

**Authors:** Kate Lewis, Maria Peppa, Bianca De Stavola, Pia Hardelid, Ruth Gilbert

**Affiliations:** 1 UCL Great Ormond Street Institute of Child Heath, London, United Kingdom

## Objectives

We provide a national overview of survival to primary school and recorded special educational needs (SEN) provision among children with hospital identified major congenital anomalies (MCAs) born in England. We also report changes before and after government reform of SEN in 2014.

## Methods

We created a cohort of 6,180,400 singleton children born in England between 1 September 2003 and 31 August 2013 using linked administrative health and education records (from the ‘ECHILD’ database). MCAs were identified using hospital admission and mortality records during infancy. We used at least one record of SEN in state-school records as a proxy for SEN provision. We quantified: survival to age 5 using Kaplan-Meier survival analysis; the prevalence of recorded SEN during primary school Years 1 to 6; and the difference in proportion of children with recorded SEN in Year 1 before and after the 2014 government SEN reforms.

## Results

Children with any MCA had 5-year survival rates of 95.1% (95% confidence interval, CI, 95.0, 95.2), compared with 99.7% (95% CI 99.7, 99.7) among children without a MCA. 41.5% (75,202/181,328) of children with an MCA attending state-school between Year 1 and 6 had any recorded SEN compared with 25.6% (1,282,979/5,008,624) of children without a MCA. Of the 12 system-specific MCA subgroups, children with chromosomal, nervous system and eye anomalies had the largest prevalence of recorded SEN. The prevalence of recorded SEN decreased by 4.9% (95% CI -5.3, -4.4) for children with any MCA compared with a reduction of 4.3% (95% CI -4.4, -4.2) for children without a MCA, when comparing pupils in Year 1 before and after 2014.

## Conclusion

Recorded SEN among children with hospital identified MCAs was markedly higher than for those without MCAs, however more than half had no recorded SEN. Our findings suggest government reform in 2014 reduced SEN provision for children with MCAs.

